# Flexural Behavior of Engineered Cementitious Composites (ECC) Slabs with Different Strength Grades

**DOI:** 10.3390/ma18092047

**Published:** 2025-04-30

**Authors:** Fengjiang Qin, Yang Han, Xinyan Wei, Xuejun Wang, Zhigang Zhang, Xiaoyue Zhang

**Affiliations:** 1 Key Laboratory of New Technology for Construction of Cities in Mountain Area, School of Civil Engineering, Chongqing University, Chongqing 400045, China; qinfengjiang@cqu.edu.cn (F.Q.); zhangzg@cqu.edu.cn (Z.Z.); xiaoyuezhang@cqu.edu.cn (X.Z.); 2 CCCC First Highway Consultants Co., Ltd., No. 63 Keji 2nd Rd., High-Tech Zone, Xi’an 710068, China

**Keywords:** ECC slabs, flexural behavior, ductility, experimental investigation, simplified method

## Abstract

Engineering Cementitious Composites (ECC) has gained significant attention in civil engineering due to its excellent tensile strength, crack width control capability, and remarkable ductility. This study examines the influence of the ECC strength and reinforcement on the flexural behavior of ECC slabs through four-point flexural tests. The results demonstrate that ECC is well suited for flexural applications. During flexural tests, the fibers within the ECC provide a bridging effect, allowing the ECC in the tensile zone to sustain a load while developing a dense network of fine microcracks at failure. This characteristic significantly enhances the crack resistance of ECC slabs. Despite the relatively low flexural capacity of unreinforced ECC slabs, they achieve 59.2% of the capacity of reinforced ECC slabs with a reinforcement ratio of 1.02%, demonstrating the potential for using unreinforced ECC in low-load-bearing applications. Further findings reveal that high-strength ECC (HSECC) not only improves the flexural capacity of unreinforced ECC slabs but also maintains excellent ductility, enabling a better balance between the load-bearing capacity and deformation ability. However, while reinforcement enhances both the flexural capacity and energy absorption, an excessively high reinforcement ratio significantly compromises ductility. Additionally, this study proposes a simplified calculation method for the flexural capacity of ECC slabs based on the axial force and moment equilibrium, providing theoretical support for their design and application. Due to their excellent flexural behavior, ECC slabs exhibit significant potential for use in flexural components such as bridge deck slabs and link slabs in simply supported beam bridges. With continued research and optimization, their application in engineering practice is expected to become more widespread, thereby improving the cracking resistance and durability of concrete structures.

## 1. Introduction

Concrete is recognized as a crucial composite construction material, noted for its high compressive strength, extensive application, and versatility in shaping. However, its inherent weaknesses, including low tensile strength, brittleness, and limited crack resistance, have contributed to various forms of defects in concrete structures. Over the past few decades, extensive research has focused on enhancing the material properties of concrete by incorporating randomly distributed polymer fibers into the cement matrix. This has led to the development of various high-ductility cement-based materials, which have been employed as alternatives to conventional concrete to improve the structural performance of traditional concrete members [1,2,3,4]. One representative material among these is engineered cementitious composites (ECC) [5,6].

ECC exhibit large tensile strain, exceeding 3%, which is several hundred times greater than that of ordinary concrete [7]. Under tensile stretching, fine microcracks measuring less than 100 μm in width become visible [8]. Importantly, the water permeability and chloride diffusion coefficients of these microcracks are nearly identical to those found in uncracked concrete structures. This characteristic significantly enhances the damage resistance of ordinary concrete structures and improves their long-term durability [9]. Moreover, ECC exhibit a unique self-healing capability to autonomously repair microcracks, thereby preventing the ingress of corrosive substances and the corrosion of internal reinforcements [10]. Leveraging these advantages, ECC can replace ordinary concrete in engineering structures, such as bridge decks, ECC–concrete composite beams [11,12,13,14], structural reinforcements [15], and seismic-resistant structures [16,17,18], thereby effectively enhancing the overall behavior. Guan et al. [19] investigated the transverse flexural behavior of reinforced ECC slabs in a bridge deck. The results revealed that reducing the protective layer thickness of the steel bar and increasing the reinforcement ratio could enhance the load-bearing capacity and cracking resistance of the slabs. Another study by Wang et al. [20] investigated the flexural behavior of ECC slabs reinforced with high-strength stainless steel wire ropes (HSSSWR-ECC), revealing that HSSSWRs significantly improved the crack resistance, that the flexural capacity increased with the longitudinal reinforcement ratio (0.26% to 0.48%), and that the incorporation of a thickener enhanced the crack width control and ductility.

The study of ECC’ flexural behavior not only reveals the mechanical responses of ECC materials under flexural conditions but also provides an important theoretical foundation and practical guidance for the design of ECC–concrete composite flexural members. To date, numerous scholars have conducted research on the flexural behavior of ECC beams. Ding et al. [21] tested the flexural behavior of steel-reinforced ultra-high-performance engineered cementitious composite (UHP-ECC) beams via four-point flexural tests. The test results revealed that the failure of the UHP-ECC specimens occurred alongside numerous closely spaced fine cracks, which delayed the development of detrimental surface cracks. The load-bearing capacity of unreinforced UHP-ECC beams was already comparable to that of concrete beams with a reinforcement ratio of 1.86%. Additionally, the reinforced UHP-ECC beams exhibited superior stiffness, load-carrying capacities, crack control abilities, and ductility compared to reinforced concrete beams. ECC beams demonstrated two modes of failure: crack localization and the gradual strain hardening of the reinforcing steel. Shao [22] investigated the flexural failure paths and load reduction mechanisms of reinforced ECC beams. The test results indicated that the load reduction in reinforced ECC beams was primarily caused by the loss of the fiber-bridging capacity or ECC crushing. The two failure paths in reinforced ECC beams were influenced by the reinforcing ratios and steel type, resulting in different failure drift capacities.

Xu [23] conducted three-point flexural tests on steel-reinforced UHTCC beams and developed theoretical equations to calculate the moment of inertia for fully cracked sections of RUHTCC beams. The results demonstrated that UHTCC beams exhibited excellent flexural behavior, regardless of the loading mode (single-point loading or symmetrical loading). Yuan et al. [24] employed finite element method simulations to analyze the flexural processes of steel-reinforced ECC beams. The simulation results revealed that the reduction in the elastic modulus after ECC cracking had a minimal impact on the strength and stiffness of reinforced ECC beams. The tensile strength and compressive ductility of the ECC matrix significantly influenced the deformation capacity of reinforced ECC beams.

Lion [25] performed four-point flexural tests on ultra-high-strength rebar-reinforced engineered cementitious composite (ECC) beams to devise a deflection curve prediction model for these reinforced beams. Their research illustrated that ECC could collaborate effectively with ultra-high-strength rebars to efficiently control crack progression. The ultra-high-strength rebar-reinforced ECC beams exhibited commendable ductility and deformation capacities. Georgiou et al. [26] conducted four-point flexural tests on 18 reinforced ECC beams. The results showed the role of fibers in the reinforced ECC beams as crack-bridging elements and transmitters of tensile forces in the tension zone, and they were able to inhibit the lateral expansion of the beams in the compression zone.

Ge et al. [27] investigated the flexural behavior of ECC beams reinforced with a hybrid combination of FRP and steel bars. The results revealed that the hybrid reinforcement increased the flexural capacity of the beams by up to 37% compared to beams solely reinforced with FRP. Additionally, the hybrid reinforcement offered enhanced control over beam deformation and crack development. Shanour [28] investigated the flexural behavior of reinforced ECC beams using ECC materials with polyviny1alchol fibers (PVA) and polypropylene fibers (PP). The test results indicated that ECC materials had a more pronounced impact on the behavior of beams with lower reinforcement ratios. Meng [29] examined the influence of stirrups on the flexural behavior of reinforced ECC beams. The results demonstrated that ECC beams without stirrups still displayed the characteristic flexural damage mode while maintaining a good load-carrying capacity and deformation capabilities.

In addition to the aforementioned studies, research on the bending performance of ECC link slabs offers valuable insights. Lepech and Li [30,31] demonstrated that ECC link slabs exhibit superior ductility, narrower crack widths, and reduced steel reinforcement requirements compared to conventional concrete, leading to their successful application in a bridge in Southeastern Michigan. Chu et al. [32] found that, under fatigue loading, the average crack width of ECC link slabs remained below 0.102 mm. Samani [33] conducted a comparative study on the fatigue flexural performance of ECC versus self-consolidating concrete (SCC) link slabs. The results demonstrate that ECC link slabs maintain higher residual strength and energy absorption capacities even under elevated fatigue stress levels, while also exhibiting extended service lives. Hossain et al. [34] reported that, after 1,000,000 loading cycles at 40% of the ultimate load, ECC link slabs retained 91% of their deformation capacity and 93% of their energy absorption capabilities, with damage manifesting primarily as microcracking.

In conclusion, ECC demonstrate excellent tensile properties, ductility, and crack control capabilities when applied to flexural members. However, the current research primarily focuses on the influence of the reinforcement ratio and steel strength on the flexural behavior of normal ECC. There is a lack of studies on the flexural behavior of high-strength ECC slabs, particularly in comparing high-strength and normal ECC slabs, as well as reinforced and unreinforced variants. This paper investigates the flexural behavior of ECC slabs with varying strengths and reinforcement ratios through four-point flexural tests, providing a comprehensive analysis of the load–displacement curves, ductility, energy absorption capacity, and strain. Furthermore, a simplified approach is developed to estimate the flexural bearing capacity of ECC slabs.

## 2. Experimental Program

### 2.1. Slab Specimen Design

**Figure 1:**
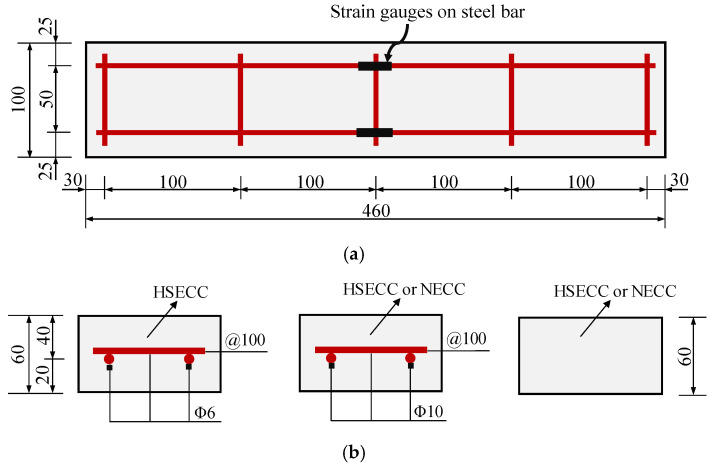
The geometry of the specimens (unit: mm): (**a**) top view; (**b**) cross-section.

Five groups of ECC slab specimens, each consisting of three specimens, were designed, with variations in their reinforcement ratios and ECC types. The dimensions of the ECC slabs were 460 mm × 100 mm × 60 mm, as shown in Figure 1a. As illustrated in Figure 1b, the influence of the reinforcement ratio and the presence or absence of reinforcement on the flexural behavior of ECC slabs with different strengths was studied by varying the reinforcement configuration. Table 1 presents the specific parameters for each specimen group. In the specimen name, “NE” represents a normal ECC, “HE” indicates a high-strength ECC, “S” designates a steel bar, and the number specifies the main reinforcement bar diameter.

### 2.2. Test Specimen Casting and Instrumentation

**Figure 2:**
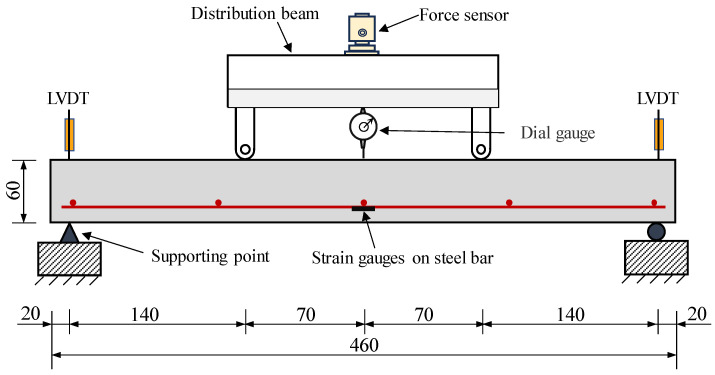
Layout of strain and deformation monitoring points (unit: mm).

**Figure 3:**
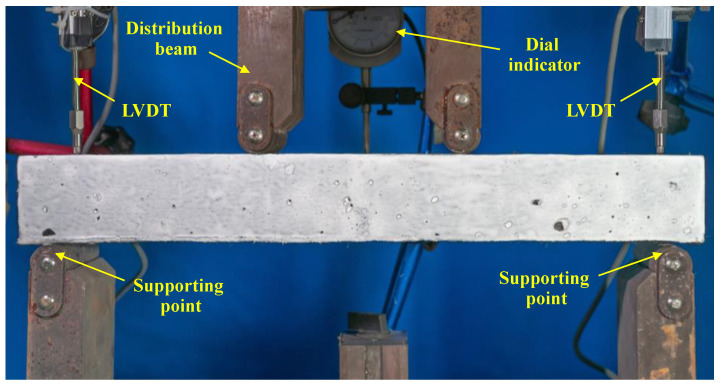
The test loading setup.

This study conducted four-point bending tests to evaluate the flexural behavior of ECC slabs. Figure 2 and Figure 3 illustrate the test setup employed in the study. The test used simple support constraints with a span of 420 mm and applied symmetrical loads at a distance of 140 mm on the specimens using a servo-hydraulic testing machine. The load was applied using displacement control at a rate of 0.5 mm/min. To measure the midspan displacement, a dial gauge was positioned in the mid-span of the slab. The settlements at the supports were measured by LVDTs. Moreover, strain gauges were placed at the bottom of the steel bars in the mid-span to monitor the stress variation in the steel bars during the flexural process.

### 2.3. Material Properties

**Figure 4:**
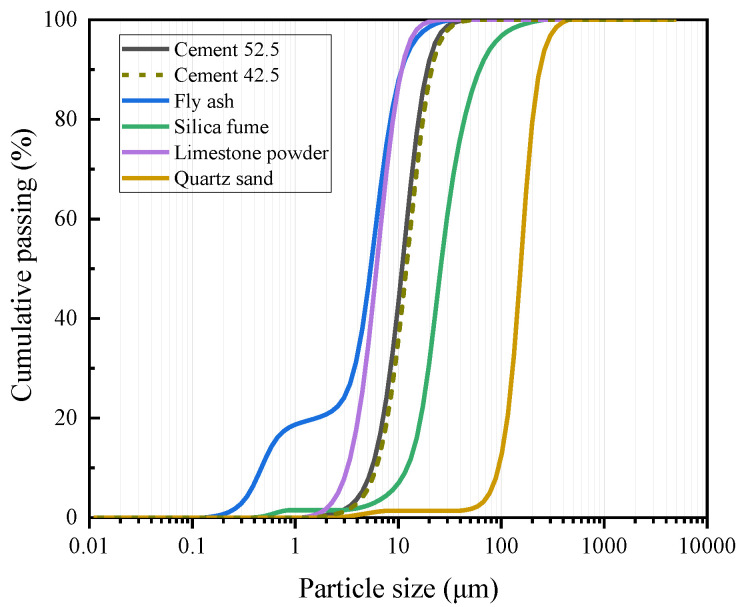
Particle size distributions of the components.

In order to investigate the effects of different strength grades of ECC on the flexural behavior of ECC slabs, a normal ECC (NECC) and high-strength ECC (HSECC) were used in this study. Cement (CEM-42.5/52.5), fly ash, silica fume, limestone powder, quartz sand, water, a water reducer, and PE fibers were used and formulated according to the mix proportions in Table 2. The dimensions and properties of the PE fiber are listed in Table 3. The chemical compositions of the materials are listed in Table 4, while their particle size distribution is illustrated in Figure 4.

Uniaxial compressive tests were performed to evaluate the compressive properties of the HSECC and NECC. Cylindrical specimens with a height of 300 mm and a diameter of 100 mm were prepared. The test procedures for the determination of the modulus of elasticity and compressive strength followed Standard GB/T 50081-2019 [35]. For each mix proportion listed in Table 2, at least three specimens were tested.

**Figure 5:**
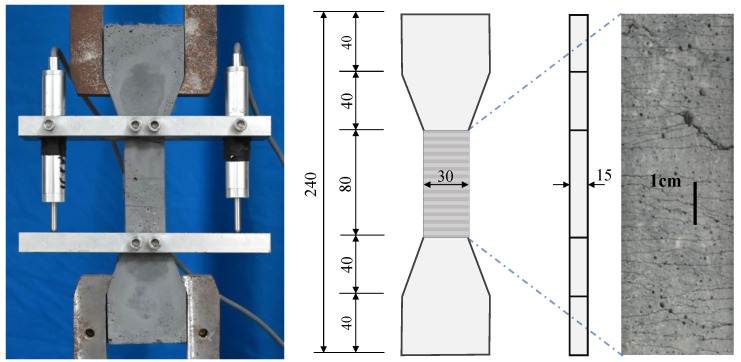
Direct tensile test specimens and test sets (unit: mm).

To evaluate the uniaxial tensile properties of the HSECC and NECC, a direct tensile test was performed. Figure 5 illustrates the dimensions of the dogbone-shaped specimens and the test setup. A universal testing machine was used to perform the tests, applying a loading rate of 0.5 mm/min. Displacements in the central regions of the specimens were recorded, along with the corresponding tensile loads. The number of cracks was determined by visually observing the damaged specimens. The average crack spacing was calculated by dividing the length of the displacement collection area (80 mm) by the number of cracks.

**Figure 6:**
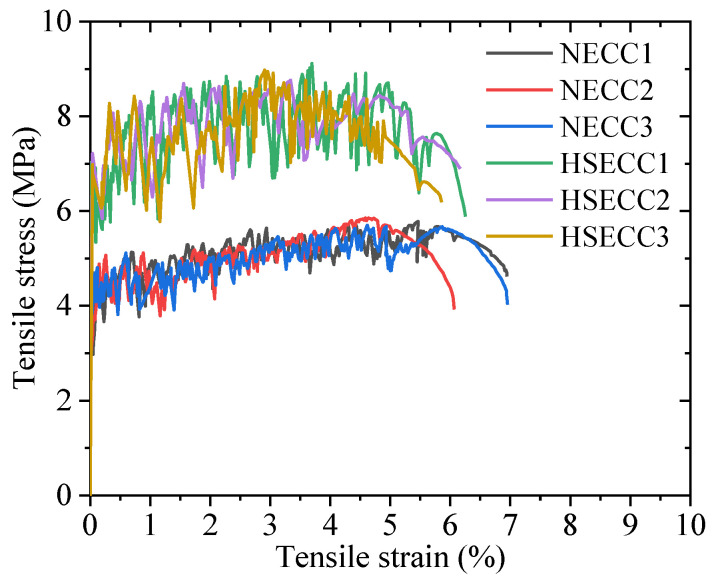
Typical stress–strain curves of ECC under uniaxial tension.

Figure 6 shows the tensile stress–strain curves of the HSECC and NECC. The ECC’s response to uniaxial tension can be characterized by two main stages. The first stage was the linear stage, beginning from the start of loading until the stress reached the initial crack strength of the ECC. The second stage was the strain-hardening stage. The fibers played a bridging effect as new cracks continued to form, allowing the ECC to sustain tensile stress until it reached its ultimate tensile strength. As illustrated in Figure 6, the fluctuation frequency of the HSECC curve is significantly lower than that of the NECC curve. This observation indicates that, under tensile loading, the HSECC develops wider crack patterns with a reduced density compared to the NECC [36]. The crack distribution of the damaged specimens is shown in Figure 5; the cracks were fine and uniformly distributed. The average crack spacing was 1600 µm. Additionally, both types of ECC exhibit good compressive performance [37]. The material properties of the two types of ECC are shown in Table 5. The longitudinal bars had diameters of 10 mm and 6 mm, and the material properties of the steel rebars are presented in Table 6.

## 3. Experimental Results and Discussion

### 3.1. Crack Distribution and Failure Mode

**Figure 7:**
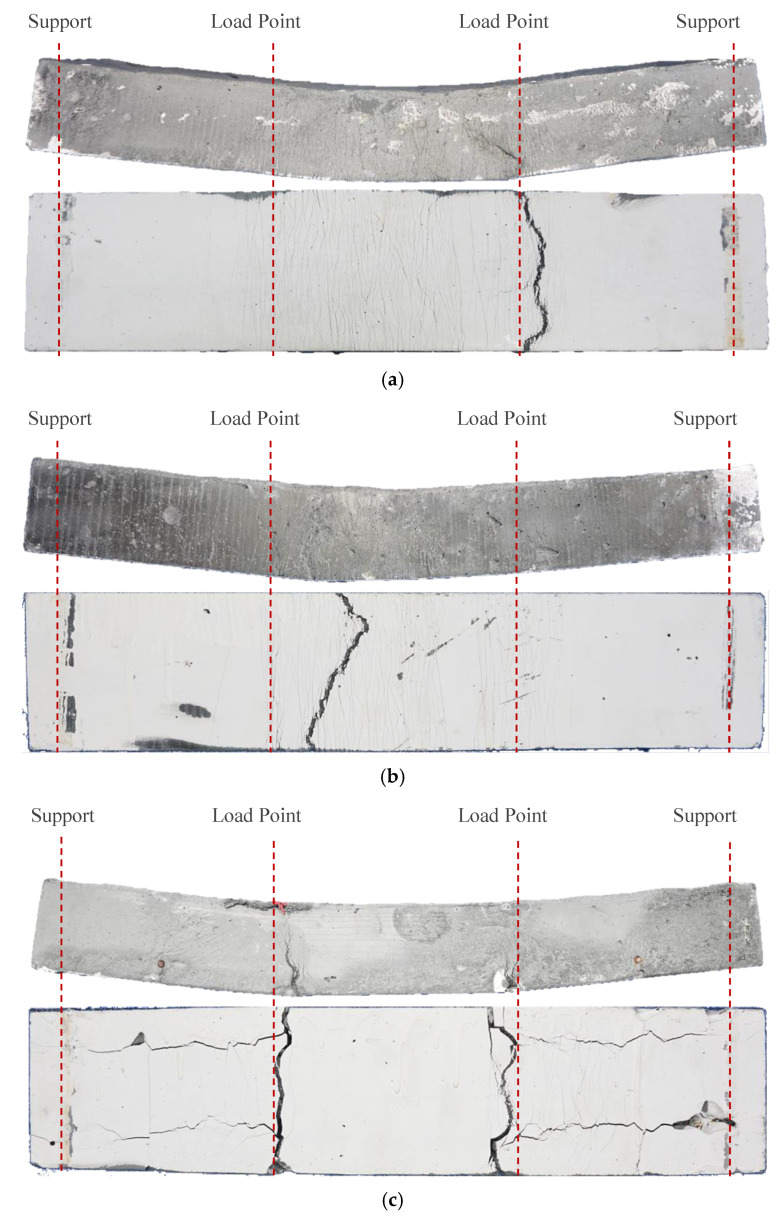

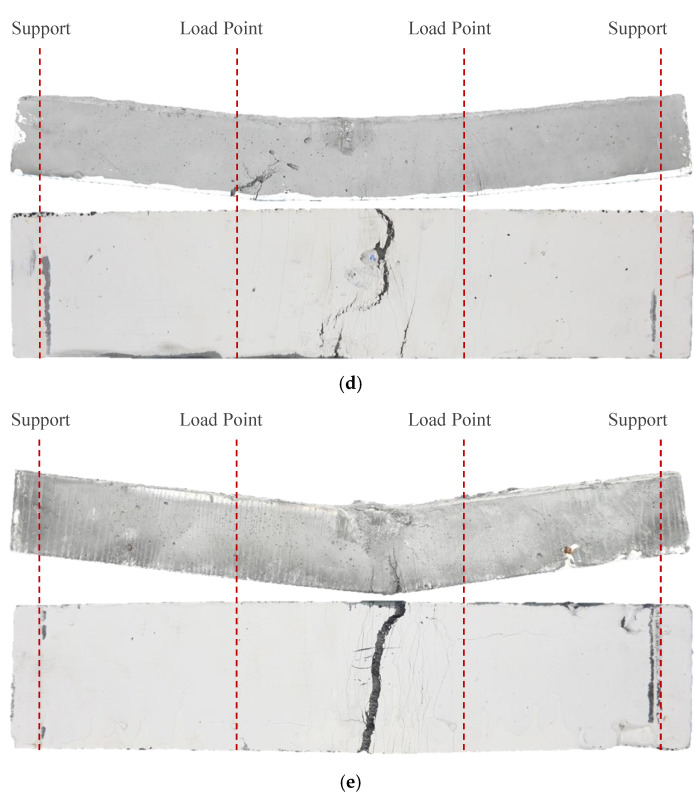
Typical failure modes of specimens (unit: mm): (**a**) NES0; (**b**) HES0; (**c**) NES10; (**d**) HES6; (**e**) HES10.

As shown in Figure 7, numerous microcracks and a main crack appear in the tensile zones of the ECC slabs, while the ECC in the compression zone of HES10 is crushed. During the flexural process of ECC slabs, when the load reaches the cracking load, the ECC at the tensile edge cracks first. However, due to the bridging effect of the fibers in the ECC, the width of the initial crack is restrained, and the fibers transfer the bridging stress to the uncracked portion of the cement matrix. As the load increases, other parts of the tension zone reach their cracking strain, and new cracks progressively form. The number of microcracks continues to rise until the fibers fracture, and the width of the most vulnerable microcracks increases. Finally, the failure mode of a wide main fracture accompanied by many microfractures in the tensile zone is formed. Compared with ECC slabs with reinforcement, there are more microcracks in the tensile areas of ECC slabs without reinforcement. The NES0 specimen has the largest number of microcracks, while the HES10 specimen has the least. This is because the longitudinal tensile bars in the reinforced ECC slab share part of the tension, reducing the tensile contribution of the ECC in the tension area, thus weakening the multi-crack cracking effect of the ECC and resulting in a decrease in the number of cracks and ductility. Overall, the multi-cracking behavior and superior ductility of the ECC material reported in [38] are also observed in the flexural members of this study, particularly in the reinforced components.

### 3.2. Load–Displacement Relationship

**Figure 8:**
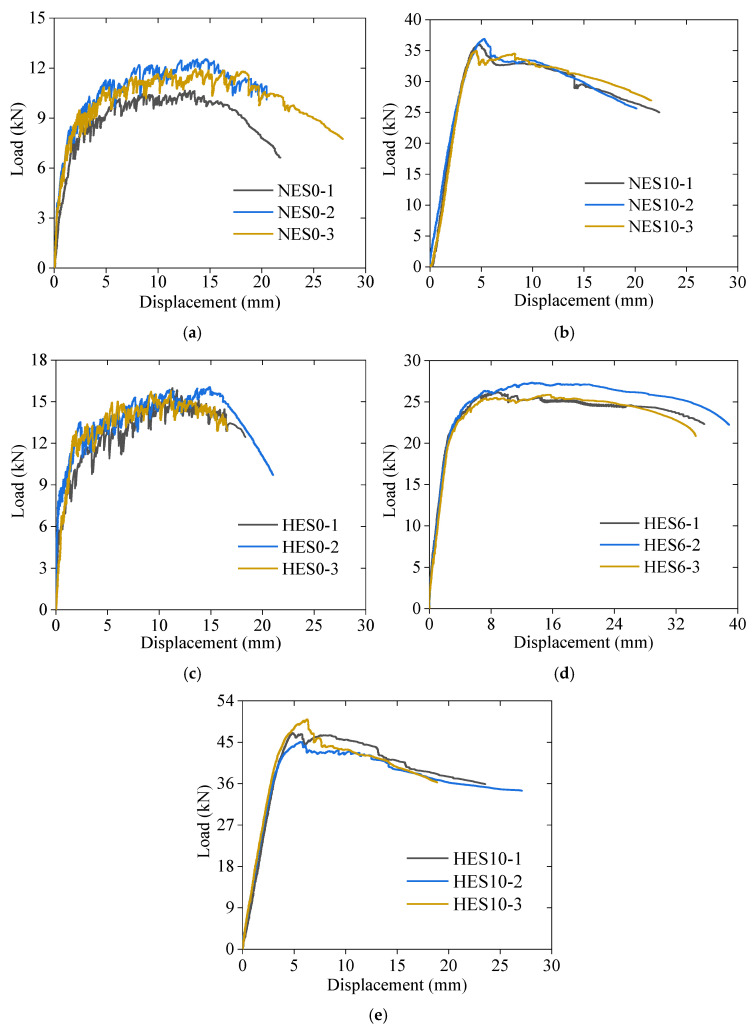
Load–displacement relationship: (**a**) NES0; (**b**) NES10; (**c**) HES0; (**d**) HES6; (**e**) HES10.

Figure 8 shows the load–midspan displacement curves for each specimen, which are categorized into three stages. In the first stage, namely the elastic phase, the specimen remains uncracked, and the midspan displacement increases linearly with the load. Once cracking occurs, the curve enters the second stage, where the stiffness decreases, leading to a faster displacement growth rate. As the load continues to increase, the curve transitions into a distinct nonlinear stage, characterized by a rapid increase in displacement. Compared with the reinforced ECC slabs, the stiffness of the unreinforced ECC slabs continuously decreases during loading, and the ascending section of the curve exhibits pronounced nonlinear characteristics. After failure, the load drops rapidly. Since NES10, HES6, and HES10 are reinforced with steel bars in the tension zone, the steel bars help to mitigate load fluctuations during ECC cracking and share part of the tensile load. As a result, the stiffness of the reinforced ECC slabs decreases more gradually during loading, and the ascending section of the curve maintains a clear linear trend. Furthermore, when the ECC in the compression zone of the reinforced ECC slabs is crushed, the presence of rebars effectively slows the rate of load reduction. The higher reinforcement ratio in NES10 and HES10 enhances the flexural capacity of the ECC slab but simultaneously reduces its ductility.

**Figure 9:**
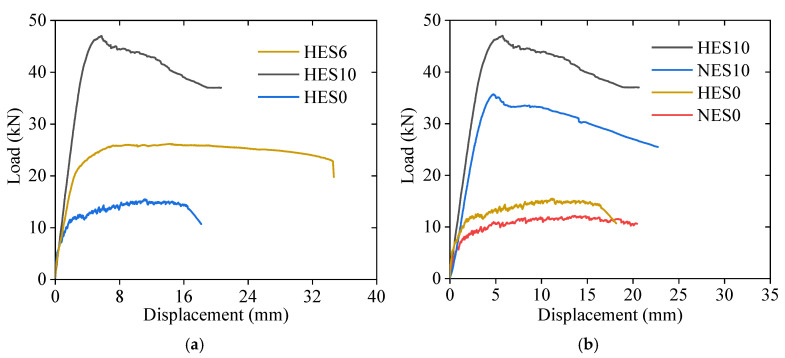
Comparison of load–displacement curves with different parameters: (**a**) reinforcement ratio; (**b**) type of ECC.

Figure 9 presents a comparison of the load–displacement curves for specimens with different parameters. This analysis investigated the impact of the reinforcement ratio and ECC strength on the flexural behavior of the ECC slabs. The average results of three identical specimens from each group were considered. As shown in Figure 9a, steel bars greatly enhance the flexural capacity of ECC slabs. The HES6 slab achieved an ultimate load of 26.2 kN, representing a 69% increase compared to the HES0 slab (15.5 kN). Furthermore, the HES10 slab reached an ultimate load of 47.0 kN, representing a 79% increase compared to the HES6 slab (26.2 kN), with a corresponding ultimate displacement of only 5.8 mm, which was merely 41% of the ultimate displacement achieved by the HES6 slab (14.3 mm). These results highlight that steel-reinforced ECC slabs, with an appropriate reinforcement ratio, can demonstrate high load-carrying capacities and excellent deformation capacities. A similar trend was observed in the flexural behavior of the ECC members reported in [21]. Upon examining Figure 9b, it is clear that the HES0 slab outperforms the NES0 slab, with an ultimate load of 15.5 kN, representing a 28% increase compared to the NES0 (12.1 kN) slab. The ultimate displacement of the HES0 slab measured 11.2 mm, while that of the NES0 slab was 14.8 mm, both significantly higher than the ultimate deformation of normal concrete flexural members. Although the bearing capacity of an unreinforced ECC slab is lower, it can still reach 59.2% of the bending capacity of a reinforced ECC slab with a reinforcement ratio of 1.02%. For slabs with low bearing capacity requirements, ECC can be constructed without reinforcement. This demonstrates that unreinforced ECC slabs offer a greater bearing capacity and flexural deformation resistance compared to conventional concrete slabs.

Table 7 summarizes the cracking load *F*_c_, yield load *F*_y_, ultimate load *F*_u_, yield displacement ∆_y_, and ultimate displacement ∆_u_, as obtained from Figure 9. The yield point was determined using the graphical method [39]. On the load–midspan displacement curve, a horizontal line is drawn through the ultimate load point, while a secant line is constructed at 75% of the ultimate load. The displacement corresponding to their intersection is defined as the yield displacement, and the corresponding load on the curve is identified as the yield load.

The failure behavior of the HES10 and NES10 slabs was similar, except that the HES10 slab exhibited a higher load-carrying capacity. Table 7 shows that the HES10 slab had an ultimate load of 47.0 kN, surpassing that of the NES10 (35.6 kN) slab by 32%. Similarly, the ultimate displacement of the HES10 slab was 5.8 mm, indicating a 21% increase compared to the NES10 slab (4.8 mm). It is worth noting that increasing the ECC strength significantly affects the load-carrying capacity when the reinforcement ratio is higher, but its impact on the deformation capacity is minimal.

### 3.3. Ductility Analysis of Slabs

Ductility is the ability of a structure to deform inelastically without a significant reduction in its load-carrying capacity, and it is an important design requirement in reinforced concrete structural design. The ductility of a structure ensures its ability to deform plastically, avoiding brittle damage and severe damage under dynamic loading. According to GB 50010-2010 (in Chinese) [40], the formula for the calculation of the ductility index is as given below:

(1) $$\mu =\Delta_{u}/\Delta_{y}$$

To assess the ductility of ECC slabs, the ductility index values for each group specimen were calculated using the data from Table 7. The ductility index values of HES0 and NES0 are close to each other. This shows that HSECC can not only improve the flexural capacity but also maintain excellent ductility. Moreover, the ductility index of the HES6 slab, with a reinforcement ratio of 1.02%, remained unchanged compared to the HES0 slab. However, the ductility index values of the HES10 and NES10 slabs with 2.85% reinforcement are only 1.6 and 1.3, which are very close to each other. This indicates that, while a higher reinforcement ratio can effectively enhance the flexural capacity, it significantly reduces the ductility.

**Figure 10:**
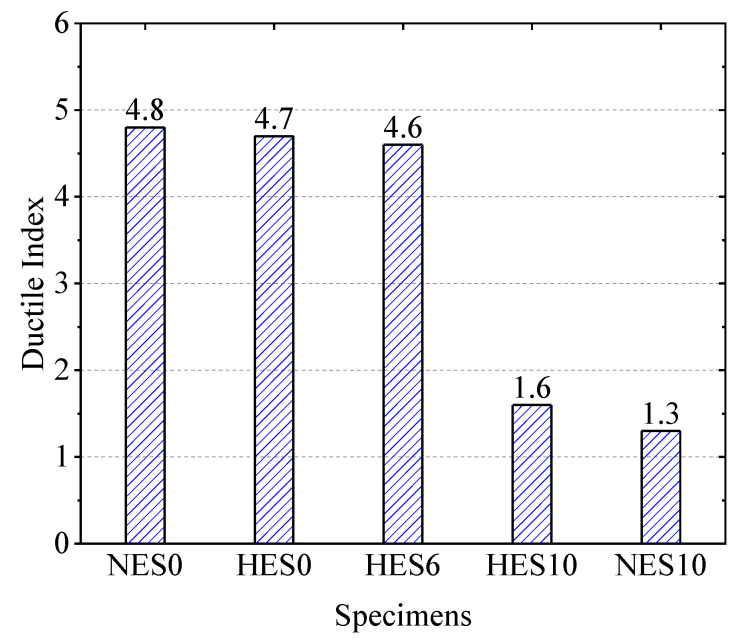
Ductility indices of the specimens.

Figure 10 demonstrates the variation in the ductility indices of the specimens. When the reinforcement ratio increased from 0 to 1.02%, the ductility indices of the specimens remained relatively consistent. However, as the reinforcement ratio continued to rise to 2.85%, the ductility factor decreased significantly. This observation aligns with the trend of the reinforcement ratio’s impact on the ductility of ECC slabs without stirrups recorded in the literature [21]. At this stage, there was no significant difference in the ductility index between the HES10 and NES10 slabs. These findings indicate that the ductility of unreinforced ECC slabs primarily depends on the material properties. Conversely, in reinforced ECC slabs, the reinforcement ratio becomes the primary factor influencing the ductility.

### 3.4. Energy Absorption Capacity

**Figure 11:**
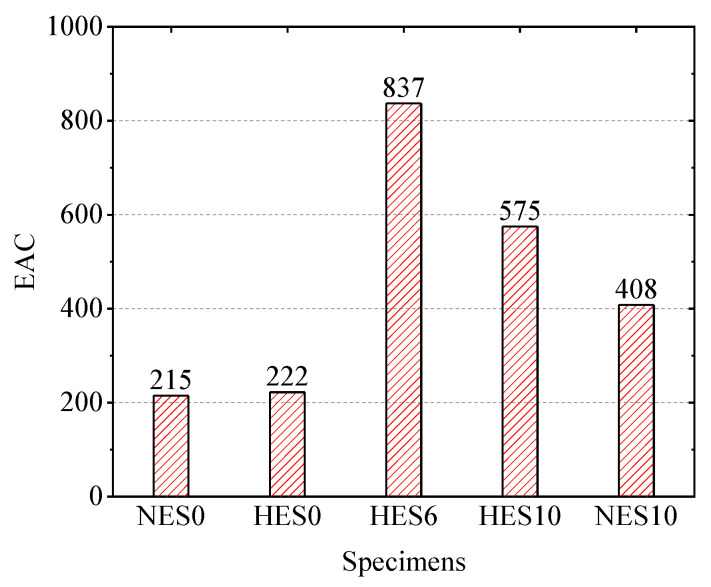
Energy absorption capacities of the specimens.

The energy absorption capacity (EAC) is a crucial structural characteristic, determined by calculating the enclosed area in the load–displacement curve when the load decreases to 0.85 times the ultimate load [11]. Figure 11 presents the energy absorption capacities of each specimen group. The HES0 and NES0 slabs exhibited similar energy absorption capacities. However, for a reinforcement ratio of 2.82%, the HES10 slab demonstrated an energy absorption capacity of 575, which was 41% higher compared to the NES10 slab (408). The use of steel bars led to the minimum doubling of the energy absorption capacity for the ECC slabs, with the HES6 slabs showing a four-fold improvement. Nonetheless, when the reinforcement ratio increased from 1.02% to 2.82%, the energy absorption capacity decreased. These observations highlight that the energy absorption capacity is influenced by a combination of the load-bearing capacity and deformation. The introduction of steel bars and the enhancement of the ECC strength significantly enhanced the energy absorption capacity of the ECC slabs.

### 3.5. Load–Strain Relationship

**Figure 12:**
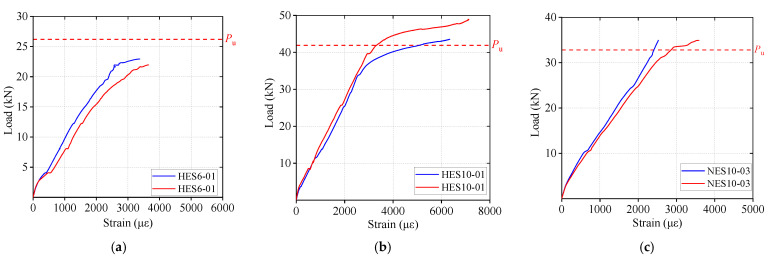
Stress–load curves of reinforced ECC slabs: (**a**) HES6; (**b**) HES10; (**c**) NES10.

Due to the damage of some strain gauges during the test, certain data were lost. To ensure the reliability and representativeness of the analysis, only two well-preserved strain–load curves were selected for each group of reinforced ECC slab specimens, as shown in Figure 12. The analysis results indicate that the steel bars in the specimens had already yielded before the reinforced ECC slabs reached flexural failure, demonstrating that the steel bars fully contributed to the load-bearing capacity throughout the loading process. Furthermore, despite the low ductility exhibited by the ECC slab with a high reinforcement ratio (2.82%) on the load–span displacement curve, the failure mode remained typical flexural failure rather than any brittle failure mechanism. This finding suggests that, even at a high reinforcement ratio, the reinforced ECC slab maintains its plastic flexural failure characteristics.

## 4. Simplified Method for Prediction of Flexural Capacity

The flexural capacity is an important index to measure the flexural behavior of a member. In this section, a simplified calculation model for the prediction of the flexural capacity of ECC slabs is established. This model references the flexural theory of reinforced concrete beams and provides a formula for the calculation of the capacity.

### 4.1. Fundamental Assumptions

Based on GB 50010-2010 [40], the strain-hardening properties of ECC, and the observed structural responses of ECC slabs in testing, several assumptions are required to estimate the flexural capacity of steel-reinforced ECC slabs. The following assumptions are proposed:

- Cross-sections perpendicular to the axis of the ECC slab, which remain planar after flexural deformation and are perpendicular to the axis of the deformed slab;
- Deformation coordination (a good bond between the ECC slab and reinforcement during loading);
- Considering the strain-hardening behavior of ECC after cracking, i.e., the ECC in the cracked area can still withstand a certain amount of tension;
- Not considering the effect of shrinkage creep of the ECC material.

### 4.2. Material Constitutive Model

**Figure 13:**
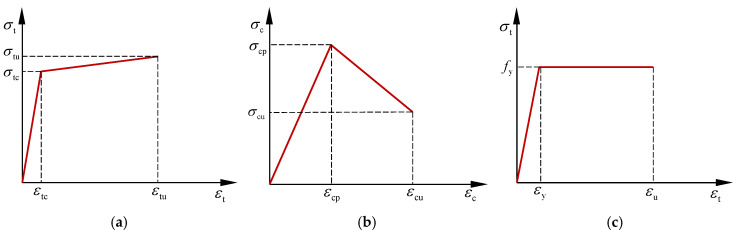
Constitutive relationships: (**a**) ECC tensile stress–strain curve; (**b**) ECC compressive stress–strain curve; (**c**) steel bar stress–strain curve.

The ECC stress–strain relationship is shown in Figure 13a,b. In order to simplify the calculation, the bifold model is used to describe the stress–strain curves of ECC materials in uniaxial tension and compression. The tension and compressive stress–strain relationship can be written as [41]

(2) $$\left\{\begin{array}{l} \sigma_{t}=\frac{\sigma_{\mathrm{tc}}}{\varepsilon_{\mathrm{tc}}}\varepsilon_{t}\;0\leq \varepsilon_{t}\leq \varepsilon_{\mathrm{tc}}\\ \sigma_{t}=\sigma_{\mathrm{tc}}+\frac{\sigma_{\mathrm{tu}}-\sigma_{\mathrm{tc}}}{\varepsilon_{\mathrm{tu}}-\varepsilon_{\mathrm{tc}}}\left(\varepsilon_{t}-\varepsilon_{\mathrm{tc}}\right)\;\varepsilon_{\mathrm{tc}}\leq \varepsilon_{t}\leq \varepsilon_{\mathrm{tu}} \end{array}\right.$$

where *ε*_tc_ and *σ*_tc_ represent the tensile strain at first cracking and its corresponding stress; *ε*_tu_ and *σ*_tu_ denote the ultimate tensile strain and the corresponding tensile stress; and *ε*_t_ and *σ*_t_ indicate the tensile strain and stress in the ECC, respectively.

(3) $$\left\{\begin{array}{l} \sigma_{c}=\frac{\sigma_{\mathrm{cp}}}{\varepsilon_{\mathrm{cp}}}\varepsilon_{c}\;\;\;\;\;\;\;\;\;\;0\leq \varepsilon_{c}\leq \varepsilon_{\mathrm{cp}}\\ \sigma_{c}=\frac{\sigma_{\mathrm{cp}}}{\varepsilon_{\mathrm{cp}}}\varepsilon_{c}+\frac{\sigma_{\mathrm{cu}}-\sigma_{\mathrm{cp}}}{\varepsilon_{\mathrm{cu}}-\varepsilon_{\mathrm{cp}}}\left(\varepsilon -\varepsilon_{\mathrm{cp}}\right)\;\varepsilon_{\mathrm{cp}}\leq \varepsilon_{c}\leq \varepsilon_{\mathrm{cu}} \end{array}\right.$$

where *ε*_c_ and *σ*_c_ indicate the compressive strain and stress in the ECC, respectively; *ε*_cp_ and *σ*_cp_ represent the peak compressive strain and stress; and *ε*_cu_ and *σ*_cu_ denote the ultimate tensile strain and the corresponding tensile stress, respectively.

The stress–strain curve of the steel bar with a distinct yield point is generally modeled as an ideal elastic–plastic model, which is shown in Figure 13c. The equation is as follows [41]:

(4) $$\left\{\begin{array}{l} \sigma_{s}=E_{s}\varepsilon_{s}\;0\leq \varepsilon_{s}\leq \varepsilon_{y}\\ \sigma_{s}=f_{y}\;\;\varepsilon_{y}\leq \varepsilon_{s}\leq \varepsilon_{u} \end{array}\right.$$

where *σ*_s_ and *ε*_s_ are the stress and strain in the steel bar, respectively; *E*_s_, *f*_y_, *ε*_y_, and *ε*_u_ are the elasticity modulus, yield strength, yield strain, and ultimate tensile strain of steel bars, respectively. *ε*_u_ = 0.01.

### 4.3. Simplified Calculation Method

The flexural damage process of reinforced ECC slabs can be delineated into three distinct stages: the no-crack stage, the working stage with cracks, and the failure stage. Initially, during the loading phase, the mid-span tensile stress remained low, keeping the bottom of the ECC slab intact, without any cracks. Upon reaching a certain load threshold, initial cracking occurred at the bottom of the ECC slab. Despite this, owing to the strain-hardening property of the ECC, it could still effectively sustain the load. As the load further increased, additional cracks emerged and extended, leading to the enlargement of the tensile zone and an rise in the reinforcement strain. Eventually, the steel bars reached the yield strain, marking the onset of the destructive stage. At this juncture, the deformation of the ECC slab cross-section escalated abruptly, while the load-carrying capacity exhibited only a marginal enhancement. This trend persisted until the ECC attained the ultimate compressive strain at the top of the slab, culminating in crushing. Conversely, in the case of unreinforced ECC slabs, the load could escalate following the initial cracking stage, as the ECC fibers in the tensile zone could bridge the cracks, thereby transferring the stresses. The unreinforced ECC slab was deemed to be in a critical state of damage when the top of the slab reached the peak compressive strain.

**Figure 14:**
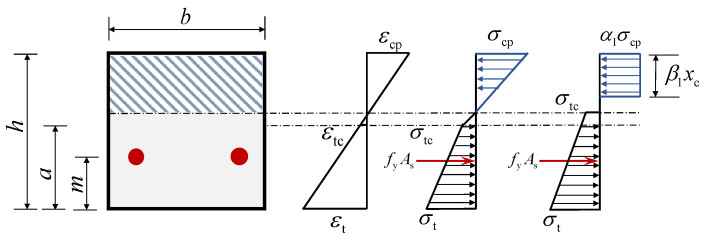
Failure mode 1.

**Figure 15:**
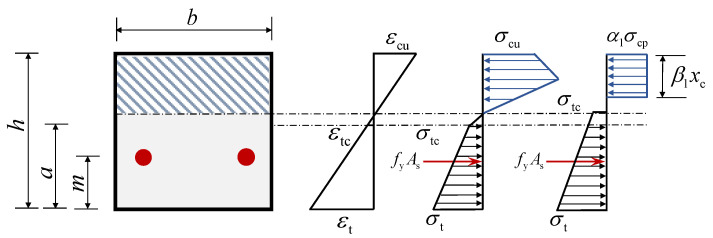
Failure mode 2.

Based on the compressive stress–strain relationship of the ECC, there are two potential scenarios for the strain in ECC in the compression zone when failure occurs: reaching either the peak compressive strain or the ultimate compressive strain. Consequently, two distinct damage states are possible. Figure 14 and Figure 15 illustrate these two critical damage states of the ECC slabs, providing a basis for calculating the flexural capacity.

Here, *h* represents the height of the cross-section, *x*_c_ is the height of the compression zone, *a* denotes the cracking strength of the distance to the slab, *m* denotes the distance from the resultant point of the steel bars to the tensile edge of the cross-section, *β*_1_*x*_c_ is the height of the equivalent compression zone, *α*_1_*σ*_cp_ is the equivalent rectangular compressive stress, and *α*_1_ and *β*_1_ denote the equivalence of the coefficients.

To simplify the calculation process, the compressive stress distribution in the ECC is approximated as a rectangular distribution. This assumption is needed to ensure that the forces are balanced and that the joint action point is the same. The stress distribution within the compressed zone can be categorized into two cases as follows.

(1) When the compressive stress in the compression zone is linearly distributed, as in Figure 14.

The force equilibrium equation and the resultant force position equation are derived in Equations (5) and (6).

(5) $$\frac{1}{2}\frac{\sigma_{\mathrm{cp}}}{\varepsilon_{\mathrm{cp}}}\varepsilon_{c}x_{c}b=\alpha_{1}\sigma_{\mathrm{cp}}\beta_{1}x_{c}b$$

(6) $$\frac{1}{3}x_{c}=\frac{1}{2}\beta_{1}x_{c}$$

(7) $$\alpha_{1}=\frac{3\varepsilon_{c}}{4\varepsilon_{\mathrm{cp}}},\;\beta_{1}=\frac{2}{3}$$

(2) When the compressive stress in the compression zone is bilinearly distributed, as in Figure 15.

Similarly, based on the equilibrium of forces and the same point of action of the resultant force, the formulas for the calculation of *α*_1_ and *β*_1_ can be derived, as shown in Equations (8) and (9).

(8) $$\beta_{1}=\frac{\sigma_{\mathrm{cp}}\left(1-\frac{\varepsilon_{\mathrm{cp}}}{\varepsilon_{c}}+\frac{\varepsilon_{\mathrm{cp}}^{2}}{3\varepsilon_{c}^{2}}\right)+\frac{\sigma_{\mathrm{cu}}-\sigma_{\mathrm{cp}}}{3\left(\varepsilon_{\mathrm{cu}}-\varepsilon_{\mathrm{cp}}\right)}\frac{{\left(\varepsilon_{c}-\varepsilon_{\mathrm{cp}}\right)}^{3}}{\varepsilon_{c}^{2}}}{\sigma_{\mathrm{cp}}\left(1-\frac{\varepsilon_{\mathrm{cp}}}{2\varepsilon_{c}}\right)+\frac{\left(\sigma_{\mathrm{cu}}-\sigma_{\mathrm{cp}}\right){\left(\varepsilon_{c}-\varepsilon_{\mathrm{cp}}\right)}^{2}}{2\left(\varepsilon_{\mathrm{cu}}-\varepsilon_{\mathrm{cp}}\right)\varepsilon_{c}}}$$

(9) $$\alpha_{1}\beta_{1}=1-\frac{\varepsilon_{\mathrm{cp}}}{2\varepsilon_{c}}+\frac{\left(\sigma_{\mathrm{cu}}-\sigma_{\mathrm{cp}}\right){\left(\varepsilon_{c}-\varepsilon_{\mathrm{cp}}\right)}^{2}}{2\sigma_{\mathrm{cp}}\left(\varepsilon_{\mathrm{cu}}-\varepsilon_{\mathrm{cp}}\right)\varepsilon_{c}}$$

In the failure state, the ECC slab experiences significant deformation. During this stage, the majority of the strain in the tensile zone exceeds the cracking strain. Therefore, the stress distribution in the tension zone can be approximated as a trapezium shape, with the initial crack stress at the neutral axis. This is illustrated in Figure 14 and Figure 15.

When the ECC slab is in mode 1 of the failure state, the strain at the upper edge of the compression zone is denoted as *ε*_cp_. According to Equation (10), the strain at the bottom of the tensile zone can be determined based on the plane section assumption, allowing for the calculation of *σ*_t_ (Equation (2)).

(10) $$\frac{\varepsilon_{t}}{\varepsilon_{\mathrm{cp}}}=\frac{h-x_{c}}{x_{c}}$$

When the ECC slab is in mode 2,

(11) $$\frac{\varepsilon_{t}}{\varepsilon_{\mathrm{cu}}}=\frac{h-x_{c}}{x_{c}}$$

the ECC slabs need to satisfy the force balance equation, as shown in Equation (12). *α*_1_ and *β*_1_ can be calculated using different formulas depending on the failure mode.

(12) $$b\alpha_{1}\sigma_{\mathrm{cp}}\beta_{1}x_{c}=f_{y}A_{s}+\frac{1}{2}\left(\sigma_{\mathrm{tc}}+\sigma_{t}\right)\left(h-x_{c}\right)b$$

The material properties of the ECC are shown in Table 8, and the yield strengths of Φ6 and Φ10 rebars are 454 MPa and 463 MPa, respectively. The theoretical bearing capacity *F*_u,th_ of the ECC slabs in the test is calculated and compared with the experimental values *F*_u,ex_. The results are shown in Table 9. The geometric dimensions of the specimens are shown in Figure 1. The average ratio of the measured to predicted flexural capacity for each ECC slab is 0.999, with a coefficient of variation of 0.08, suggesting that the accuracy of the simplified calculation method proposed in this paper is satisfactory.

## 5. Conclusions

In this study, a total of nine steel-reinforced ECC slabs and six unreinforced ECC slabs were prepared and subjected to four-point flexural tests until damage. Two reinforcement ratios and two ECC strengths were used as test variables. The failure modes, load–midspan displacement curves, ductility, EAC, and strain–load curves of the ECC slabs were analyzed. A simplified load capacity calculation model for ECC slabs was proposed. Based on the experimental and analytical results obtained in this study, the following conclusions can be drawn.

1. ECC slabs subjected to flexural deformation are characterized by multi-seam cracking and large displacement before the loss of the load-carrying capacity, which can improve the safety of the structure. In addition, harmful cracks in ECC slabs can be reduced because ECC can control the crack width well. This advantage helps to prevent the corrosion of steel bars in ECC slabs, thus improving the durability of steel-reinforced ECC members.
2. ECC slabs reinforced with steel bars can significantly improve their flexural capacity and EAC, but the ductility decreases as the reinforcement ratio increases. The flexural capacity of HES6 and HES10 is 69% and 203% higher than that of HES0, respectively. However, the ductility of HES10 is only 34% of that of HES0. Therefore, when designing ECC slabs, an appropriate reinforcement ratio should be chosen to fully leverage the combined performance of the ECC and reinforcement.
3. With the increase in the ECC strength, the bearing capacity of the ECC slab also increases, but the ductility is almost unaffected. The HSECC has higher compressive and tensile strength compared with the NECC, so it can improve the load-carrying capacity of ECC slabs. The load-carrying capacity of HES0 slabs increases by 28% compared with NES0 slabs, and the load-carrying capacity of HES10 slabs increases by 32% compared with NES10 slabs.
4. Using the equilibrium condition between the axial force and flexural moment, a simplified method for the prediction of the flexural capacity of ECC slabs is developed. The average predicted-to-experimental ratio is 1.003, with a coefficient of variation of 0.08. This method can serve as a guide for the bending design of ECC slabs.

The four-point flexural tests show that ECC slabs exhibit multiple fine cracks and large deformation due to the fiber-bridging effect, which maintains the load-bearing capacity after cracking and enhances the crack resistance. Steel reinforcement improves the flexural capacity and energy absorption capacity, although excessive amounts reduce the ductility. Higher ECC strength further increases the capacity with little ductility loss. A simplified flexural capacity calculation method based on force equilibrium provides reliable predictions. These results highlight the durability advantages and application potential of ECC slabs in bridge decks and similar structures.

## Figures and Tables

**Table 1:** Details of specimens.

Specimen	Type of ECC	Main Steel Bars	Reinforcement Ratio	Specimen Number
NES0	N-ECC	/	0	3
NES10	N-ECC	2Φ10	2.85%	3
HES0	HS-ECC	/	0	3
HES6	HS-ECC	2Φ6	1.02%	3
HES10	HS-ECC	2Φ10	2.85%	3

**Table 2:** Mix proportions of ECC mixture (unit: kg/m^3^).

Mix Ratio	CEM-52.5	CEM-42.5	Fly Ash	Silica Fume	Limestone Powder	Quartz Sand	Water	Water Reducer	Fiber
NECC	0	475	827	0	0	384	354	6	18
HSECC	750.4	0	401.2	84.9	194.5	461	260	17	22

**Table 3:** Properties of PE fiber.

Length (mm)	Fiber Diameter (µm)	Modulus (GPa)	Density (g/cm^3^)	Elongation (%)	Tensile Strength (MPa)
12	26	100	0.97	2.4	3000

**Table 4:** Chemical composition of binder materials (wt%).

Material	CaO	Al_2_O_3_	SiO_2_	SO_3_	Fe_2_O_3_	MgO	CaCO_3_	LOI
Cement 52.5	64.78	5.29	18.86	3.05	2.01	3.05	0.00	2.96
Cement 42.5	62.6	5.76	20.57	3.02	2.05	3.00	0.00	3.00
SF	0.52	0.36	97.28	0.38	0.12	0.37	0.00	0.97
FA	9.19	25.78	42.30	2.06	9.14	3.79	0.00	7.74
LP	0.00	0.00	2.31	0.00	0.00	0.00	97.61	0.00

**Table 5:** Properties of ECC (unit: MPa).

Material	Elasticity Modulus	Compressive Strength	Crack Strength	Tensile Strength	Ultimate Tensile Strain
NECC	22,200	56.2	4.3	5.6	0.06
HSECC	26,100	73.1	6.9	8.3	0.04

**Table 6:** Properties of steel bars (unit: MPa).

Material	Diameter (mm)	Elasticity Modulus	Yield Strength	Tensile Strength
HRB400	6	206,000	454	665
HRB400	10	206,000	463	673

**Table 7:** Experimental results.

Specimen	$F_{c}$ (kN)	$F_{y}$ (kN)	$\Delta_{y}$ (mm)	$F_{u}$ (kN)	$\Delta_{u}$ (mm)	μ	EAC
HES0	5.85	11.8	2.4	15.5	11.2	4.7	222
HES6	5.11	21.9	3.1	26.2	14.3	4.6	837
HES10	5.57	41.9	3.7	47	5.8	1.6	575
NES0	3.51	9.2	3.1	12.1	14.8	4.8	215
NES10	3.98	32.8	3.8	35.6	4.8	1.3	408

**Table 8:** Material properties of ECC (unit: MPa).

Material	$E_{c}$	$\sigma_{\mathrm{tc}}$	$\sigma_{\mathrm{tu}}$	$\varepsilon_{\mathrm{tu}}$	$\sigma_{\mathrm{cp}}$	$\varepsilon_{\mathrm{cp}}$ (10^−6^)	$\sigma_{\mathrm{cu}}$	$\varepsilon_{\mathrm{cu}}$ (10^−8^)
HSECC	26,100	6.9	8.3	0.04	73.1	2832	36.6	4548
NECC	22,200	4.3	5.6	0.06	52.6	2687	26.3	4931

**Table 9:** Comparison of theoretical and experimental values (unit: kN).

Specimen	*F*_u,th_	*F*_u,ex_	*F*_u,th_/*F*_u,ex_
HES0	16.9	15.5	1.09
HES6	27.4	26.2	1.05
HES10	44.0	47.0	0.94
NES0	11.0	12.1	0.91
NES10	36.0	35.6	1.01
Average			0.999
COV			0.08

## Data Availability

The original contributions presented in this study are included in the article. Further inquiries can be directed to the corresponding author.
